# Effects of the Solvent Vapor Exposure on the Optical Properties and Photocatalytic Behavior of Cellulose Acetate/Perylene Free-Standing Films

**DOI:** 10.3390/polym15132787

**Published:** 2023-06-23

**Authors:** Gustavo Coderch, Alexander Cordoba, Oscar Ramírez, Sebastian Bonardd, Angel Leiva, Marleen Häring, David Díaz Díaz, Cesar Saldias

**Affiliations:** 1 Departamento de Química Física, Facultad de Química y de Farmacia, Pontificia Universidad Católica de Chile, Macul, Santiago 7820436, Chile; gustavo.coderch@uc.cl (G.C.); acordoba@uc.cl (A.C.); ogramirez@uc.cl (O.R.); aleivac@uc.cl (A.L.); 2 Instituto Universitario de Bio-Organica Antonio Gonzalez, Universidad de La Laguna, Avda. Astrofísico Francisco Sanchez, 38206 La Laguna, Spain; sbonardd@ull.edu.es; 3 Departamento de Química Orgánica, Universidad de La Laguna, Avda. Astrofísico Francisco Sánchez, S/N, 38206 La Laguna, Spain; 4 Institut für Organische Chemie, Universitat Regensburg, Universitatsstr. 31, 93053 Regensburg, Germany; marleen.haering@chemie.uni-regensburg.de

**Keywords:** films, biobased materials, photodegradation

## Abstract

The search to deliver added value to industrialized biobased materials, such as cellulose derivatives, is a relevant aspect in the scientific, technological and innovation fields at present. To address these aspects, films of cellulose acetate (CA) and a perylene derivative (Pr) were fabricated using a solution-casting method with two different compositions. Consequently, these samples were exposed to dimethylformamide (DMF) solvent vapors so that its influence on the optical, wettability, and topographical properties of the films could be examined. The results demonstrated that solvent vapor could induce the apparent total or partial preferential orientation/migration of Pr toward the polymer–air interface. In addition, photocatalytic activities of the non-exposed and DMF vapor-exposed films against the degradation of methylene blue (MB) in an aqueous medium using light-emitting diode visible light irradiation were comparatively investigated. Apparently, the observed improvement in the performance of these materials in the MB photodegradation process is closely linked to the treatment with solvent vapor. Results from this study have allowed us to propose the fabrication and use of the improved photoactivity “all-organic” materials for potential applications in dye photodegradation in aqueous media.

## 1. Introduction

During the last two decades, organic molecules that are photoactive under visible light have been intensively studied due to their wide range of potential applications of current technological importance, such as light-emitting devices [1], sensors [2], solar cells [3], energy conversion [4], and photocatalysis [5] to name a few. In this sense, to exploit the photofunctionality of this type of molecules, some of the strategies followed are mainly based on chemical modification or blends with other organic agents having compatible or complementary optical properties [6,7]. These strategies have important implications for the absorption and emission light behaviors of the photoactive materials [8,9]. Considering these strategies, perylenes (Pr) intended for use as photoactive materials have shown considerable absorption response in the visible zone of the light spectrum [10,11]. For example, based on both their physical (solubility, optical absorption) and chemical (structure, functionality) molecular properties [12,13,14], numerous perylene derivatives have been extensively studied as dyes and paints in various industrial fields. In general, these systems exhibit an appreciable thermal and chemical stability which allows the coating or functionalization of surfaces as an example [15,16]. Additionally, at present, Pr systems stand out for their photophysical properties, such as high quantum yields, photochemical stability, and a marked electron acceptor character [17,18]. Considering the above factors, various perylene derivatives have been used in various electronic and optical applications, such as organic photovoltaic cells [19], photoelectrodes [20], and artificial photosynthesis [21]. Despite their highly desirable optical and electronic attributes, one of the main drawbacks of Pr systems (in addition to other photoactive organic molecules) is that they do not by themselves form handleable flexible films that could potentially be integrated into photosensitive multilayer circuits or arrays [22,23]. Consequently, this issue considerably limits the possibility of expanding the range of technologically important Pr applications [24]. Accordingly, when using Pr, it is desirable to achieve an appropriate chemical stability over time and ensure a continuous interfacial contact area and homogeneous distribution in the presence of other photoactive layers in addition to increasing photostability to prolong its useful life. Therefore, the incorporation of photoactive molecules into transparent polymer films could be a plausible strategy to achieve a suitable balance between the stability of photophysical properties and their cost-effective performance in a specific application [25,26]. According to this approach, various polymeric materials displaying film-forming capabilities can be obtained from different biomass resources [27,28]. In this context, cellulose derivatives emerge as ideal candidates because they are readily available, abundant, renewable, low-cost, and inert matrices that can host various types of molecules. Particularly, cellulose acetate (CA) is one of the most important cellulose esters and shows the capability to form transparent thin films under mild experimental conditions obtained from the acetylation of cellulose, a characteristic that favorably influences its solubility and processability properties [29,30]. In addition, CA in a film format turns out to be free-standing and transparent, and possesses good thermal, mechanical, and optical properties [31]. Therefore, it can be foreseen that the combination of the attributes of polymeric materials derived from biomass and the photophysical and photocatalytic properties of perylene derivatives would allow “all-organic” photofunctional materials to be obtained. 

In addition, in the literature, it has been reported that the surface morphology of films of different nature and thickness can be altered in some way by exposure to a determined solvent vapor [32,33,34]. In this way, this type of treatment has direct effects on the topographic and optical properties of the material [35]. Several authors attribute this behavior to the preferential orientation of regions or functional groups towards the environment saturated with solvent vapor [32,34,36,37]. Thereby, these types of strategies could be used to explore the above-mentioned effects on photoactive materials and their performance in specific chemical environments. 

On the other hand, numerous investigations have used high power irradiation sources (300–1000 W) to study photocatalytic processes [38,39,40]. In addition, to the best of our knowledge, the use of low power light (such as light-emitting diodes [LEDs]) in photocatalysis using polymeric films was further investigated recently [37]. The latter results in a very promising approach to use low energy sources to trigger photodegradation processes mediated by photoactive polymeric films. Therefore, the use of LED radiation sources for photocatalytic processes involving independent photoactive polymeric films is of highly scientific and technological interest. Thus, a viable and innovative strategy can be developed to utilize “all-organic” biobased, free-standing, optically transparent, and photofunctional polymeric materials for applications in the field of photocatalysis. In this work, we focused our attention on the preparation, characterization, and study of the optical and photocatalytic properties of optically transparent and free-standing CA/Pr films influenced by exposure to DMF vapor. Specifically, the properties specific for the catalytic photodegradation reaction of methylene blue were investigated under cold-white LED irradiation. Characterization of the resulting films was carried out using ultraviolet/visible (UV/vis) spectroscopy, contact angle, and atomic force microscopy (AFM) techniques. In addition, theoretical electronic calculations were conducted to obtain better insight into the geometric and energetic parameters in addition to the nature of the participating intermolecular interactions that determine and govern the optical and photocatalytic properties of CA/Pr systems.

## 2. Experimental Section

### 2.1. Materials

Cellulose acetate (39.7 wt.% acetyl content. ${\bar{M}}_{n}$ ca. 50,000) was used as received from Sigma-Aldrich, Germany. Merck S.A supplied organic solvents. Perylene derivative (purity > 98%) was kindly provided by Professor Dr. David Diaz from the Universidad de La Laguna. The water used in all experiments was purified by a Millipore Milli-Q system (resistivity higher than 18.2 MΩ cm).

### 2.2. Preparation of CA/Pr Free-Standing Films

Film samples were prepared using the solution-casting method directly from stock solutions of perylene (Pr) and cellulose acetate (CA) in acetone [41]. The concentrations of Pr and CA in the acetone solution were 1.0% *w/v* (250 mg) and 5% *w/v* (2.5 g), respectively. To achieve different Pr concentrations in the films, the mixed solutions were adjusted to contain 0.3% *w/w* and 0.8% *w/w* of Pr. After vigorous stirring for 8 h, the solutions were poured into Petri dishes and air-oven dried at 35 °C for 24 h. The resulting CA/Pr free-standing films were labeled as CA/Pr 0.3% and CA/Pr 0.8%.

### 2.3. Characterization Techniques

The film samples were analyzed using an Agilent Cary 60 spectrophotometer to record their UV/vis spectra. Water contact angles were measured using an optical contact angle apparatus (Data physics OCA20 system) equipped with a high-resolution CCD camera and SCA 20 software (Data Physics Instruments). The initial water contact angle (q0) was determined by triplicate measurements, depositing a droplet of 10.0 ± 0.2 μL on the film surface. Both the support and injecting syringe were kept at a temperature of 25.0 °C. The surface films were characterized using a 3110-Nanoscope IV atomic force microscope (AFM) in tapping mode. Film thicknesses were measured in ten random points in each sample using a handheld digimatic micrometer (Mitutoyo Corp., Kawasakishi, Japan), resulting in an approximate thickness of 25.0 μm.

### 2.4. Solvent Vapor Exposure Experiments

For these experiments, CA/Pr films were exposed at different times to saturated DMF solvent vapors in a closed container maintained at 40 °C for 6 h. After exposure to DMF vapors, the films were then placed in a vacuum oven at 30 °C for 24 h to study their optical, wettability, and topographical properties at certain time intervals. 

The optical bandgap (*E_g_*) was estimated from equation [42]:

(1) $$E_{g}=\frac{1240}{\lambda_{th}}$$

in which *E_g_* represents the optical energy bandgap (in eV), and λ_*th*_ is the threshold wavelength (in nm) at the onset of the absorption spectrum. The above mathematical expression is recognized as valid for conjugated aromatic organic systems, such as Pr.

The kinetic resulting data were processed considering a pseudo-first order mechanism with respect to the absorbance of each of the films. Consequently, the mathematical expression shown below was [43]:

(2) $$\mathrm{Ln}\;A_{t}{}_{CA/Pr}=\mathrm{Ln}\;A_{0}{}_{CA/Pr}-{k^{\prime }}_{CA/Pr}\;t$$

in which ${k^{\prime }}_{CA/Pr}$ is the apparent rate constant and $A_{t}{}_{CA/Pr}$ y $A_{0}{}_{CA/Pr}$ are the absorbance values at time t and initial time, respectively. Therefore, a plot of $\mathrm{Ln}\;A_{t}{}_{CA/Pr}\;vs\;t$ yields ${k^{\prime }}_{CA/Pr}$ as slope. 

### 2.5. Photocatalysis Experiments

The study of photocatalytic performance of the as-prepared CS/Pr polymer films was carried out under visible-light irradiation using a cold-white (λ_max_ = 447 nm and λ_max_ = 560 nm) LED illumination source (Lumileds^TM^, 3 W/m^2^) and 20 pieces of surface mounted diodes. Specifically, the evaluation of the photocatalytic activities of the CA/Pr films was performed by immersion of the respective samples (2 cm × 2 cm) into 10 mL of a 1.5 mM methylene blue (MB) aqueous solution. Once the system was prepared, it was left in the dark for 2 h until the equilibrium state between room temperature and controlled stirring was reached. Changes in the absorbance of the dye were monitored using UV/vis spectroscopy at 650 nm (the wavelength of the maximum absorption of MB). 

To investigate the eventual influence of non-exposed and exposed films on the kinetics of this reaction, an expression similar to Equation (2) was employed [44]:

(3) $$\mathrm{Ln}\;A_{t}{}_{MB}=\mathrm{Ln}\;A_{0}{}_{MB}-{k^{\prime }}_{MB}\;t$$

where $\;A_{t}{}_{MB}$ y $A_{0}{}_{MB}$ represent the absorbance values of MB that correspond to time *t* and the initial time, respectively. ${k^{\prime }}_{MB}$ is the apparent rate constant for photodegradation of MB.

## 3. Results and Discussion

**Scheme 1:**
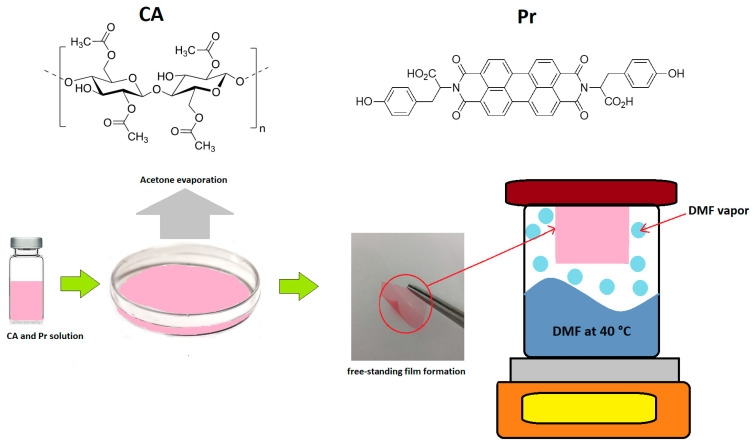
Chemical structure of cellulose acetate and perylene (CA and Pr, respectively). Illustrative representation of the fabrication of CA/Pr films and the dimethylformamide (DMF) exposure process.

Details on the experimental route used for the synthesis of the Pr can be found elsewhere [45]. Conveniently, the chemical structures of CA and Pr and the film fabrication strategy in addition to the DMF exposure process are shown in Scheme 1. It is important to mention that low Pr contents were used so as not to drastically affect the handleability of the formed CA/Pr films, an effect that currently represents a drawback with this type of polymer systems [46]. 

### 3.1. Optical Properties

**Figure 1:**
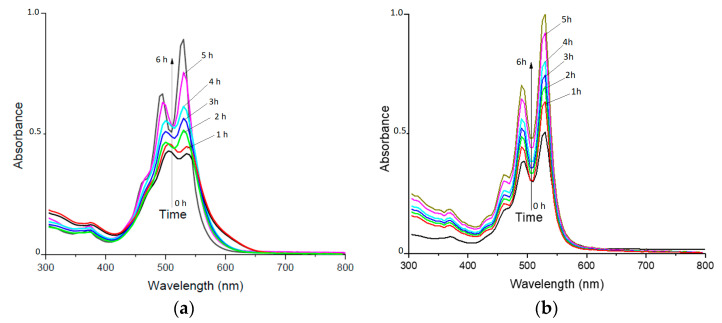
Ultraviolet/visible (Uv/vis) spectra of (**a**) cellulose acetate/perylene (CA/Pr) 0.3% and (**b**) CA/Pr 0.8% films exposed to dimethylformamide (DMF) vapor for 6 h.

Initially, the behavior of CA/Pr films exposed to DMF vapors was studied in terms of their optical, wettability, and topographical properties. Specifically, a systematic monitoring of the different mentioned properties depending on the exposure time was conducted (Figure 1). Monitoring of the optical behavior revealed notable increases in the respective absorbance values for the samples exposed to the solvent. Initially, the spectra of the 0.3% and 0.8% CA/Pr films showed two absorption maxima at approximately 500 and 560 nm in addition to a shoulder at approximately 460 nm. These bands would be consistent with the characteristics of transition energies of the type S0 → S1 (e.g., 0–0, 0–1, and 0–2). Interestingly, in both cases, the absorbance of the film samples exposed to DMF vapors increased markedly over the measured time interval. In the case of the 0.3% sample, it could be observed that as the exposure time to DMF vapor increased, a slight shift toward blue occurred, which could be attributed to the change in the environment of the Pr entities. Presumably, the vicinities of each of these molecules are gradually enriched with larger domains of aromatic rings and electron donor–acceptor functional groups. This process could be attributed to the change in the ratio of the absorbances of the bands at 560 and 500 nm that strongly depend on the spatial distance between these molecules. For the CA/Pr 0.8% sample, very slight redshifts were detected since increasing the concentration of Pr molecules in the polymer matrix favors an environment that is abundant in electrons from aromatic systems. The gradual increase in absorbance by virtue of exposure time to DMF vapor suggests that the probability of electronic transitions increases due to changes in the micropolarity, microviscosity, and dielectric attributes of the surrounding chemical environment of the Pr molecules. In this sense, the functional groups of the CA polymer could also contribute to this behavior by orienting toward the DMF vapor-saturated medium after a certain time interval.

The *E_g_* values (Equation (1)) for the CA/Pr 0.3% and 0.8% films were 2.0 and 2.2 eV, respectively. As expected, the presence, chemical structure, and concentration of Pr in the CA matrix contribute to the processes of charge mobility and electron transfer in the solid phase, which helps explain the values of the optical bandgap values for each of the samples.

**Figure 2:**
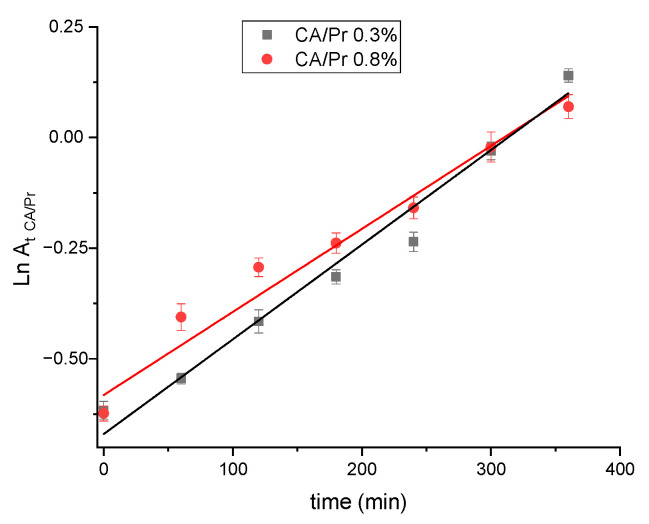
Rates of increase in absorbances of CA/Pr 0.3% (black line) and CA/Pr 0.8% (red line) films exposed to DMF vapor after fitting to a pseudo-first order model.

Complementarily, kinetic studies based on absorbance changes over time due to exposure to DMF vapors were conducted (Figure 2). The values of ${k^{\prime }}_{CA/Pr}$ (Equation (2)) for CA/Pr 0.3% y CA/Pr 0.8% are 1.78·10^−3^ ± 7.1·10^−4^ s^−1^ y 2.07·10^−3^ ± 5.4·10^−4^ s^−1^, respectively. Apparently, the fit of the kinetic data along with the estimated values of rate constants would corroborate that the process of change in the optical properties of the films would be the responsibility mainly of a single species, in this case Pr. This finding agreed with the absorbance changes observed mainly in the visible region of the spectra.

### 3.2. Wettability Properties

**Figure 3:**
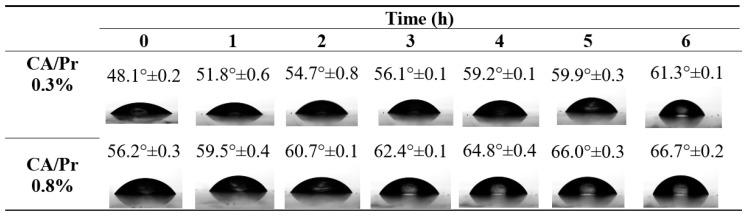
Contact angle profiles and values (N = 3) for the samples for the samples CA/Pr 0.3% and CA/Pr 0.8% at different times of exposure to DMF vapors.

Contact angle measurements using water were conducted to study the changes in the surface properties of the CA/Pr films in terms of the hydrophilic/hydrophobic balance that were induced by the exposure time to DMF vapors. Figure 3 shows the profiles and contact angle values (θ) for CA/Pr as a function of exposure time. In both cases, a gradual increase in the values of θ over time could be observed. This phenomenon could be explained because the spreading of the drop on the surface of the film prevails in the initial stage (at t = 0, prior to exposure to DMF vapor) since these regions would be enriched in more hydrophilic groups originating from the matrix of CA. Note that the value of this angle is larger for the CA/Pr 0.8% sample, which could be attributed to the higher concentration of Pr in the polymeric matrix, which gives this material a more marked hydrophobic character. Additionally, as the exposure time progresses, the phenomenon of adsorption of water drops on the film’s surface becomes predominant. It is likely that the response exhibited by the perylene molecules present in the polymer matrix is mediated by their selective interaction with DMF, which promotes a change in the surface morphology of the films. This change could be triggered by preferential orientation of the chemical skeleton or functional groups of the Pr toward the polymer–solvent vapor interface during the exposure period. 

### 3.3. Topographical Properties

**Figure 4:**
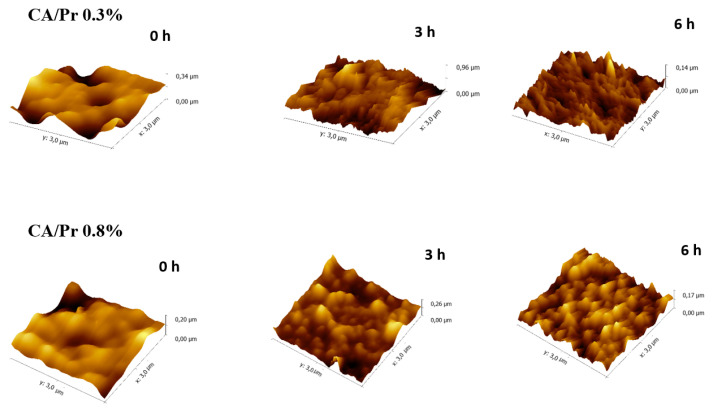
(3 × 3 μm^2^) Atomic force microscopy (AFM) images of CA/Pr 0.3% and CA/Pr 0.8% at different times of exposure to DMF solvent vapor.

AFM images at different times of exposure to DMF vapors of CA/Pr films are shown in Figure 4. For both samples prior to exposure, a relatively smoother surface and relatively homogeneous topography could be observed. After 3 h of exposure, the topography undergoes a noticeable change, which was mainly demonstrated by the increase in surface roughness. At the end of the exposure time, the surface roughness increased significantly, thus accounting for the formation of microaggregates or microphases. These detected variations were consistent with the root mean square (RMS) roughness values of each of the samples at different exposure times (Table 1). Specifically, the RMS values tended to increase for both systems as the exposure time progressed. These observations are consistent with the mentioned above phenomena since they would account for the selectivity of the DMF vapor toward the Pr entities susceptible to being promoted to rearrangements and reorganization at the micro-nanoscale level in the polymeric matrix surface. Note that the Pr concentration also plays a role in the surface morphology. This finding was confirmed since the CA/Pr 0.3% and CA/Pr 0.8% films show a morphology of the microaggregates that tend to be more rounded and sharper, respectively, after 6 h of exposure. In this sense, the 0.8% concentration sample confirms a larger amount of Pr entities that are organized toward the DMF polymer–vapor interface, conditioning the appearance of smoother or accentuated shapes that are determinant in the roughness of the surface.

### 3.4. Photocatalysis Studies

**Figure 5:**
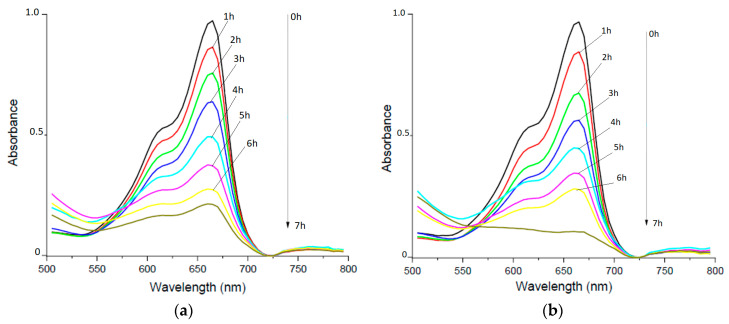
Spectrophotometric monitoring of methylene blue (MB) photodegradation, mediated by CA/Pr 0.8% films (**a**) not exposed and (**b**) exposed to DMF vapor. Note that white LED light was used to carry out these processes.

To examine the photocatalytic properties of the prepared films, the degradation of MB was monitored as a model reaction in the presence of the CA/Pr 0.8% sample before and after exposure to solvent vapor. This reaction was selected because numerous reports in the literature are available in which different perylene derivatives act as photomediators for the degradation of methylene blue using light radiation. Figure 5 displays the monitoring of the photodegradation process using a white LED light of MB mediated by the presence of the CA/Pr film before and after exposure to DMF vapors. In both cases, the decrease in the band centered at 650 nm could be clearly noticed over time (for a total of 7 h of monitoring). 

**Figure 6:**
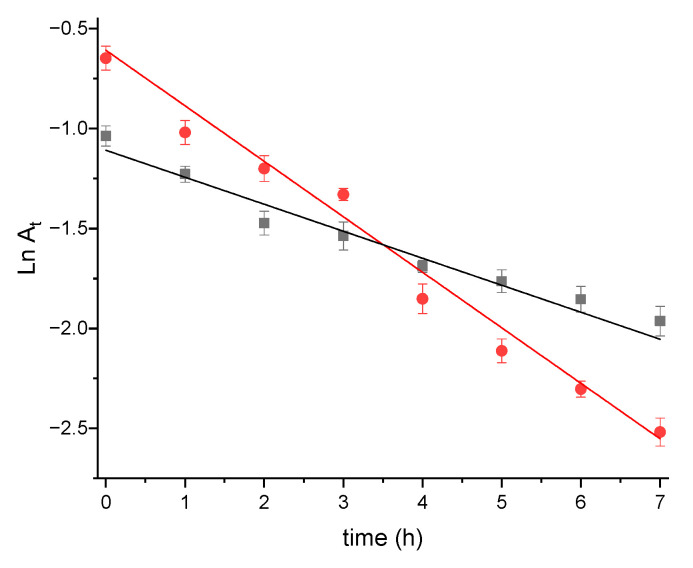
MB photodegradation rates mediated by CA/Pr 0.8% films unexposed (black line) and exposed (red line) to DMF vapor.

Figure 6 shows the plots of $\mathrm{Ln}\;A_{t}{}_{MB}\;vs\;t$ from which ${k^{\prime }}_{MB}\;$ can be estimated considering the slopes (Equation (3)) in both cases (namely, films exposed and not exposed to DMF vapor). Accordingly, the values of ${k^{\prime }}_{MB}$ for non-exposed and exposed CA/Pr were 0.13 ± 0.010 s^−1^ and 0.27 ± 0.019 s^−1^, respectively. Interestingly, the value of the constant is approximately two times for the exposed film with respect to the non-exposed film. It is likely that the change in the optical properties (such as the increase in absorbance) of the film after exposure to DMF vapor influences the photocatalytic performance in MB degradation. Apparently, these acquired attributes for the exposed system would help improve light-mediated processes. Although no pronounced shifts in the absorbance wavelength for each of the experiments were detected, the increases in absorbance for the exposed films can be related to a certain increase in the electronic transition probability, such as in the n–π* types. Eventually, this type of transition would be the main contributor to its behavior for the most efficient absorption of light. Note that the same reaction was tested in the absence of the CA/Pr systems under white light irradiation. Additionally, the reaction was performed in the presence of the CA/Pr systems under dark conditions. In both experiments, it was confirmed that the reaction does not proceed under the conditions described above since no significant changes were detected at 650 nm for MB in the UV/vis absorption spectrum. Consequently, the changes that were detected in the surface properties of the films after their exposure to DMF vapor could play an important role in the catalytic photodegradation process of MB. As an example, it was observed that the roughness of the films increased significantly after treatment with DMF vapor. The variation in this parameter was reflected by the increase in the number of active sites at which the MB adsorption–desorption phenomena occur onto the surface of the CA/Pr film. Thus, alterations in both the initial optical and surface properties upon the solvent vapor treatment would result in advantageous cooperative phenomena for the photocatalytic degradation process of MB.

On the other hand, the photoactivity and reuse of CA/Pr 0.8% films exposed or not exposed to DMF vapor were preliminarily evaluated during four consecutive cycles of MB photodegradation reaction. For this evaluation, the films were washed and rinsed with Milli-Q water after each use cycle. The photocatalytic activity (PA) of the films was estimated using Equation (4) [45]:

(4) $$PA\%=\frac{k_{x}}{k_{1}}\times 100;x=1,\;2,\;3\;and\;4$$

in which *k_x_ and k_1_* correspond to the MB photodegradation rate of each of the cycles after use and the photodegradation rate of the 1st cycle, respectively.

**Figure 7:**
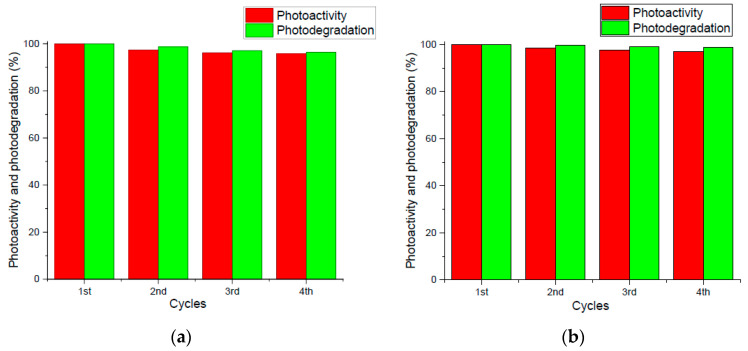
The photodegradation of MB and the photoactivity of CA/Pr 0.8% films (**a**) not exposed and (**b**) exposed to DMF vapor upon each cycle of use.

Importantly, Figure 7 shows that the reusability and photoactivity of the non-exposed films and DMF vapor-exposed films in the MB photodegradation process did not experience a severe decrease in their respective performances after each cycle. This process is a very relevant aspect since the untreated and DMF-treated free-standing “all-organic” films showed a relatively constant performance for the number of cycles to which they were subjected. This process would help confirm that the DMF vapor treatment of CA/Pr 0.8% films does not drastically affect their reusability in the photodegradation of MB in aqueous medium. Presumably, the mechanism that prevails in the photodegradation process would consist of three consecutive stages: (1) adsorption of MB on the surface of the film, (2) photodegradation induced by light (such as appearance and intervention of reactive oxygen species [ROS]), and (3) desorption of the species that result as products of photodegradation. According to these antecedents, the presence of Pr along with DMF vapor treatment of CA/Pr films provides a viable strategy for improving the efficiency and activity of the MB photodegradation catalytic process. It is important to highlight that these types of systems offer explorable alternatives for scaling-up due to their versatility, potentially low toxicity for use in bodies of water, low-cost fabrication, high availability of components, and easy processability for light-induced chemical reactions of technological importance. In this way, these types of photoactive materials would be emerging alternatives for photodegradation of a wide variety of organic dyes in bodies or aqueous media.

## 4. Conclusions

The presented route is a relatively simple method for the fabrication of free-standing “all-organic” CA/Pr photoactive films containing low amounts of a Pr. Consequently, the optical, wettability, and topographical properties of these films were convincingly modified by exposure to DMF vapors. The change in these properties were found to influence the photocatalytic performance of the films against MB dye degradation using a white LED light. Interestingly, the systems exposed to DMF vapors reached a higher MB photodegradation rate value compared with those that were not exposed. Additionally, the photoactivity and reusability of both non-exposed and exposed films in the photodegradation of MB were not drastically affected during each of the four cycles to which they were subjected. Finally, this study exposes a scalable and comparatively economically viable strategy (organic materials only) for potential applications in photodegradation of organic dyes in water bodies.

## Figures and Tables

**Table 1:** Root-mean-square roughness (RMS) values determined by AFM for CA/Pr 0.3% and CA/Pr 0.8% films, at different times of DMF vapor exposure.

	RMS (nm)		
Film	0 h	3 h	6 h
CA/Pr 0.3%	0.8	1.7	2.5
CA/Pr 0.8%	1.1	1.4	2.0

## Data Availability

The data presented in this study are available upon request from the corresponding author.
